# Enhancing engagement in HIV care among adolescents and young adults: A focus on phone-based navigation and relationship building to address barriers in HIV care

**DOI:** 10.1371/journal.pgph.0002830

**Published:** 2025-01-09

**Authors:** Harriet Fridah Adhiambo, Chanda Mwamba, Jayne Lewis-Kulzer, Sarah Iguna, Gladys Moraa Ontuga, Dorothy Imbuka Mangale, Everlyne Nyandieka, James Nyanga, Isaya Opondo, Joseph Osoro, Lina Montoya, Edwin Nyagesoa, Norton Sang, Eliud Akama, Elizabeth Bukusi, Lisa Abuogi, Elvin Geng, Zachary Arochi Kwena

**Affiliations:** 1 Research Care Training Program, Centre for Microbiology Research, Kenya Medical Research Institute, Kisumu, Kenya; 2 Department of Child, Family, and Population Health Nursing, University of Washington, Seattle, Washington, United States of America; 3 Centre for Infectious Disease Research in Zambia, Lusaka, Zambia; 4 Department of Obstetrics, Gynecology and Reproductive Sciences, University of California San Francisco, San Francisco, California, United States of America; 5 Department of Medicine, Washington University in St. Louis, St. Louis, Missouri, United States of America; 6 Department of Biostatistics, University of North Carolina at Chapel Hill, Chapel Hill, North Carolina, United States of America; 7 Department of Global Health, University of Washington, Seattle, Washington, United States of America; 8 Department of Pediatrics, University of Colorado, Denver, Colorado, United States of America; PLOS: Public Library of Science, UNITED STATES OF AMERICA

## Abstract

Structural, psychological, and clinical barriers to HIV care engagement among adolescents and young adults living with HIV (AYAH) persist globally despite gains in HIV epidemic control. Phone-based peer navigation may provide critical peer support, increase delivery flexibility, and require fewer resources. Prior studies show that phone-based navigation and automated text messaging interventions improve HIV care engagement, adherence, and retention among AYAH. However, little is known about AYAH experiences utilizing electronic phone-based peer navigation and automated text messaging (E-NAV). We assessed the experiences of AYAH receiving phone-based peer navigation to address barriers to HIV care engagement and viral suppression. We purposefully selected participants randomized to E-NAV within the Adapt for Adolescents in Kisumu, Kenya, and conducted 20 in-depth interviews. Interviews were conducted by a trained qualitative researcher between October and December 2021 and explored topics such as health-seeking and care experiences, E-NAV acceptability and benefits, and the client-navigator relationship. The interviews were audio-recorded and transcribed. We then applied inductive and deductive coding, followed by thematic analysis. Overall, participants found E-NAV acceptable in regard to content and frequency–particularly the opportunity to select a preferred time for calls/text messages, including evenings and weekends. They found the tone of navigator calls and messages friendly, supporting relationship building. Further, AYAH-navigator relationships were described as fraternal, client-focused, and confidential, which supported a personal connection and trust. Reported E-NAV benefits included adherence and appointment reminders, increased knowledge about HIV care, and strategies to address HIV stigma. Electronic navigation is a promising method for youth peer navigation because it optimizes reach (both in time and space) for youth that have severe constraints on both while preserving the ability to create a rapport and a relationship with patients.

## Introduction

The rise of electronic communication mechanisms has opened the door to countless innovations in public health that promise to extend the reach, timeliness, and appropriateness of interventions for health. In particular, for populations such as adolescents and young adults living with HIV (AYAH), who face social (e.g., stigma) as well as structural (e.g., lack of transport) barriers, electronic engagement with health care providers could help systems meet people halfway through flexible and nimble interventions. Such interventions are a priority as gaps in engagement in HIV care and treatment among AYAH persist despite substantial progress in HIV epidemic control over the last three decades [1–3]. It is estimated that approximately 1.75 million adolescents live with HIV, with new infection rates among adolescent girls being six times higher than those among boys [4]. In Kisumu County, which has the second highest HIV prevalence in Kenya at 17.5%, adolescents accounted for 20% of the 4,012 new HIV infection infections in 2017 [4–6].

Across all the age categories of people with HIV, AYAH experience worse health outcomes due to poor adherence to antiretroviral treatment (ART) and treatment interruptions. This results in high viral load, gaps in retention and subsequently increased morbidity estimated at 3.1 deaths per 100-person years among AYAH [7, 8]. The recommended optimal adherence level for ART to be effective is 95% yet recent evidence indicates that adherence among AYAH may be as low as 65% [9, 10].

Peer support and navigation is a strategy that provides psychosocial support to AYAH and helps bridge gaps in HIV care and treatment, and is ripe for innovations using electronic and mobile platforms [11, 12]. Navigation has shown promise in improving overall population health outcomes by addressing barriers to engagement in HIV care, such as transportation, stigma, and disclosure [13–15]. However, peer support has traditionally been delivered in person at clinics or the community, which is resource-intensive and dependent on AYAH availability. Phone-based peer support—whether using text messages, social media or other platforms—may require less resources, increase the reach of delivery, enhance the appropriateness (through more privacy as compared to an in-person visit), and flexibility in timing, while maintaining critical psychosocial and treatment support leading to improved HIV care outcomes. However, there are differences in how AYAH and AYA without HIV engage with electronic communication. AYAH are more likely to use some platforms for managing their condition, accessing health information, medication reminders, and seeking peer support to minimize stigma [16]. In contrast, AYA without HIV typically use these platforms for social, recreational and general wellness purposes [17].

The rapid rise in the use of mobile phones and access to social media has improved communication and delivery of information globally. These advancements have inspired many new health promotion innovations, but to date, their potential in relation to peer navigation in Africa has not extensively been examined [18, 19]. Existing studies have focused on examining feasibility, acceptability, and efficacy of phone-based peer navigation are most frequently based in the United States [20, 21]. Social media platforms such as Facebook, Twitter, and Instagram have successfully delivered HIV care and treatment-targeted preventive messages, but less often as an adjunct to a peer relationship [22, 23]. Some emerging work that marries electronic communications with peer relationships are emerging, for example, a study conducted in Nigeria found that using social media platforms such as Facebook and WhatsApp with peer navigation led to increased uptake of HIV services, linkage and retention [24]. Despite these promising interventions, the burden of HIV among AYAH is still disproportionate.

While mobile phone access in Kenya is high (about 98% among adults and approximately 70% among youths), [25] little is known about the experiences of AYAH utilizing integrated phone-based peer navigation for HIV treatment support in low- and middle-income countries. To document experiences with phone-based peer navigation among AYAH and provide recommendations for the future design of tailored navigation interventions, we qualitatively explored the experiences with phone-based peer navigation among a subset of AYAH as part of a randomized trial to improve viral suppression and retention in Kenya [26].

## Materials and methods

### Study design

We used a qualitative approach to explore AYAH experiences with electronic peer navigation (E-NAV) among a subset of participants randomized to the E-NAV arm in the Adaptive Strategies for Preventing and Treating Lapses of Retention in Adolescent HIV Care (A4A) Study [26]. This approach was selected to yield in-depth, rich, contextual, and participant-centered insights into the impact of E-NAV on retention [27]. The ongoing A4A study examines the comparative effectiveness of adaptive strategies for engaging AYAH treatment to prevent initial engagement lapses (defined as missed clinic visits and virologic failure viral load≥200 copies/ml) and to provide intensified interventions for lapses that occur.

### Setting

This study was conducted in three public health facilities in Kisumu County, Kenya. These facilities provide HIV care and treatment services to adolescents at youth-friendly centers supported by the Kenyan Ministry of Health with technical support from an implementing partner: Center for International Health, Education, and Biosecurity (CIHEB). Antiretroviral treatment (ART) is provided free of charge to all people living with HIV. Available psychosocial support varies by facility and includes clinic-based support groups, peer educators, and case management for those with adherence and retention challenges.

### The intervention

In the main trial (A4A study), interventions were tailored using participatory approaches that included human centered design, a discrete choice experiment and focus group discussions [28, 29]. Participants are initially randomized to one of two lower intensity interventions (stage 1: E-NAV—a phone-based peer navigation arm delivered through peer navigators who were AYAH between the age of 19 and 28 years living with HIV who received a 5-day standardized training on ART adherence counseling, psychosocial support, case management, electronic and in-person navigation, data collection, and entry, or standard of care). Patients in stage 1 who miss their clinic appointments by 14 days and are not virally suppressed are re-randomized to one of three more intense interventions (stage 2: Conditional cash transfer (CCT), In-person navigation (IP-NAV), or Standard of care and intensified counselling (SOC-IC) to treat lapses in retention. Participants are followed up for two years for the primary outcome of viral suppression and retention in HIV care and treatment [26].

The E-NAV intervention (a combined strategy involving automated SMS and electronic peer navigation support through the use phone calls & social media) consists of an initial in-person meeting followed by bi-weekly sessions for the first two months and subsequently transitioning to monthly sessions. These sessions, which take place over the phone by a peer navigator, aim to address barriers and strengthen resilience among AYAH. The intervention is delivered for one year following study enrolment or until participants are re-randomized to a stage 2 intervention, whichever is sooner. E-NAV participants also receive bi-weekly automated tailored text messages focused on health promotion, visit attendance, and medication adherence. Peer navigators communicate with participants electronically on WhatsApp, Messenger, short message service (SMS), or through a phone call. This is based on the participant’s preference for the method of communication. Each peer navigator, who was not gender specific, had a caseload of approximately 20 participants. Participants are assigned to navigators who had the least caseloads at the time. The facility-based peer navigators are supervised by a Navigator Coach, with counseling experience, and trained in providing psychosocial support and HIV education targeting ART and clinic attendance adherence.

### Sampling and sample size

In this sub-study, we selected 20 participants among 314 randomized AYAH to the E-NAV arm who had received the intervention for at least six months. Adolescents were defined as individuals between ages 14 and 19 whereas young adults were defined as individuals between ages 20 and 24. Interview participants were selected from all three study clinics between October and December 2021. We used maximum variation sampling [30], a variant of purposive sampling, to select the participants. This sampling method was aimed at selecting participants to gain a diverse and deep understanding of perspectives from different sociodemographic segments (age, gender, marital status, occupation, and parity). Once potential participants were identified, the qualitative research assistant scheduled in-person interviews with the participants at a convenient time. All the participants approached agreed to participate in the interviews. Our sample size was 20 participants which was reached based on information saturation from the participant’s experiences with E-navigation that we were investigating.

### Data collection

We developed and used an interview guide to gain a deeper understanding of the experiences, nuances, and effectiveness of the E-NAV arm of the study from the participants perspective. Major topics explored included health-seeking and care experiences, acceptability and benefits of the E-NAV intervention, participants’ relationship with the phone navigator and satisfaction with the intervention. The interviews were conducted by a trained qualitative research assistant with over five years of experience working with AYAH. The guide was available in three languages: English, Kiswahili, and the local Dholuo language, and interviews were conducted in the participant’s preferred language. All 20 interviews were audio-recorded with permission from the participants and uploaded to a password-protected computer accessible to the qualitative study team. The interviews were conducted at a convenient time and place for the participants either at the clinic or in the community and each lasted approximately 60 minutes.

### Data management and analysis

Audio files were transcribed directly to English by the Research Assistants who collected the data. Transcription was done in tandem with data collection. Upon completion, the qualitative research team (ZK and CM, and SI) met to develop the initial codebook. Each team member read 5 transcripts to familiarize themselves with the data as they generated initial codes. The codes identified were discussed, reviewed, and refined during the weekly meetings to reach a consensus. The final codebook was then imported into Dedoose software (Cloud based qualitative and quantitative analysis software developed by the Sociocultural Research Consultants (SCRC), Los Angles) used to code the remaining transcripts. We used thematic analysis approach as described by Braun and Clarke [31] to identify emerging themes and sub-themes related to AYA experiences with the electronic navigation arm of the study. Theme selection was done based on the nature of the discussion and frequency in the data set.

### Ethical considerations

We obtained ethical approval to conduct this study from the institutional review boards of the Kenya Medical Research Institute (KEMRI; #3986) in Kenya and Washington University in St. Louis (WUSTL; #202006141), Missouri USA. We received assent and parental/caregiver consent from participants below 18 years of age, whereas those above 18 years provided informed consent. These interviews were conducted in a private room within the health facility or the study office.

## Results

Out of the 20 AYAHs receiving electronic navigation interviewed, 10 (50%) were female, and 13 (65%) were aged between 20–24 years. A total of 9 (45%) of the respondents had completed a secondary level of education. Furthermore, 5 participants reported being employed, 6 unemployed, and 9 were students. The majority 14 (70%) of the participants were single and 7 participants (44%) who were all females reporting having at least one child **(Table 1)**.

**Table 1:** Participant socio-demographic characteristics.

Participant Sociodemographic Characteristics		Age (Years)		Total
		15–19 count (%)	20–24 count (%)	count (%)
Gender	Male	4 (40)	6 (60)	10 (50)
	Female	3(30)	7(70)	10 (50)
Education Level	Primary	3(43)	4(57)	7(35)
	Secondary	5(56)	4(44)	9(45)
	College	1(25)	3(75)	4(20)
Occupation	Employed	0	5(100)	5(25)
	Unemployed	2(33)	4(67)	6(30)
	Student	7(78)	2(22)	9(45)
Marital status	Married	0	6(100)	6(30)
	Single	9(64)	5(36)	14(70)
Number of children	1	0	3(100)	3(42)
	2	0	2(100)	2(29)
	3>	0	2(100)	2(29)

**Fig 1:**
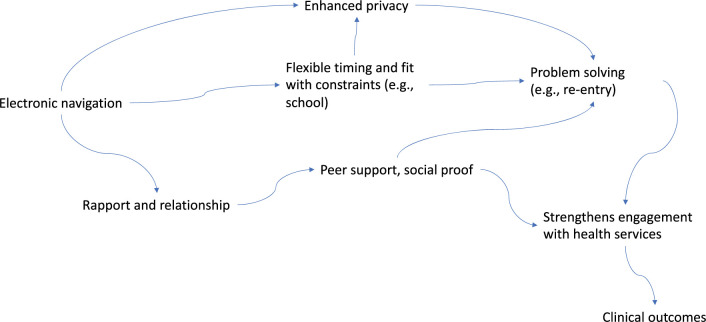
Mechanisms of action of phone-based peer navigation.

The major themes identified among AYAH who received E-NAV focused on the beneficial aspects of the intervention including intervention acceptability, ability to self-navigate health care, relationship and trust building, and HIV knowledge. There were no observable differences in perspectives of the AYAH on the E-NAV intervention based on their sociodemographic characteristics. These thematic areas demonstrate how phone-based peer navigation works. We illustrate the mechanisms by which phone-based peer navigation serves as an adjunct to HIV care and treatment demonstrating its potential to enhancing clinical outcomes (Fig 1).

### Acceptability of electronic navigation intervention

There was a high acceptability of the intervention among the participants. They reported their acceptability of the electronic navigation activities based on the timing, frequency, tone of voice and the content of the intervention.

#### Timing of the intervention

The majority of the participants reported liking that they were given the opportunity to select their preferred time for text messages and calls. Furthermore, several AYAH preferred to receive calls/text messages on weekends or weekday evenings citing busy school and work schedules. This also applied to AYAH who shared phones with caregivers who had busy schedules during the day. Choosing the preferred day and time to communicate with the navigator provided an opportunity for consistent engagement with the navigator.

*Weekdays we come from school late at times at seven, so on weekends like for today we were to come from school at five in the evening, and when I get home that’s when they call, or at times they do call on Sunday morning before I go to school at two.* ***(Female, 16- year- old, single***)

Two participants used their medication time to receive the calls and text messages from the navigator. In as much as the calls and text messages were not sent daily, they served as a reminder to take their medication.

*My timing is okay because I told them to send a message when I am almost taking my drugs, so around nine, before exactly nine that’s three or five minutes to nine they send it, so when you receive a message you have to check then you see its almost time, you go prepare so that you can take your drugs, so you can make it, to take the drugs on time.* ***(Male, 19-year-old, single***)

#### Frequency of calls and text messages

Most AYAH reported receiving text messages and calls from navigators every two weeks. They liked the frequency because the navigator could check in on them and find out how they were doing generally, if they were keeping their clinic appointments and taking their medications as prescribed. The interactions also served as an opportunity to share any challenges they were experiencing.

*The frequency was okay. When they call, they inquire about my health, how I am faring on, how I take my medication, if maybe I’m sick or I am facing any challenges. They remind me that, to make sure that I don’t miss on my clinic appointments.* ***(Female, 15-year-old, single)***

AYAHs who did not receive calls and text messages frequently cited challenges such as access to phone particularly among those sharing with caregivers and lack of electricity to charge the phones.

*If they call today, then next time will be probably the following week, when there is no power [electricity], my phone can be off.* ***(Male, 15-year-old, single***)

#### Content of the automated SMS and phone calls or messages with the Navigator

The majority of the AYAH reported that their conversations with the Navigator and the automated SMS they received were mainly focused on the importance of adhering to ART, importance of clinic attendance, and preventing HIV transmission.

*He advises me on care, on how I should be taking my medication, he advises that if I stop taking medication, I will suffer, I shouldn’t forget to take my medication, if I forget taking medication and I assume that I’ll take them the following day, that is how the virus will multiply, I tell him that I can’t stop, and I’ll continue taking my medication.* ***(Female, 20-year-old, married***)

While confidentiality is not guaranteed when using mobile phones, AYAH liked the content of the messages specifically because coded language used guarded against accidental disclosures.

*Take your snack [Not real word], its time, if you have any queries just ask us, or call on that number. Snack in this case are the drugs, the ARVs, they put it so, in that if anyone gets access to the message, they won’t understand, apart from the two who are conversing are the ones who do understand such* ***(Male, 19-year-old, single***).

*The message is confidential*, *and you can’t understand for example not everyone understands what ‘room’ [not real name] means*, *they won’t tell the ‘room’ they are referring to in the text and if they ask*, *you’ll tell them it is a certain group that I joined*, *and they won’t tell what ‘Room’ refers to* ***(Male*, *21-year-old*, *single***).

However, two AYAH who had not disclosed their status or had not informed their partners of their involvement in the study were not comfortable receiving calls from the Navigators, especially when they were with their partners or significant others. This was thought to be a potential source of unintended disclosure.

*My husband didn’t know that I am on care, so maybe they called when we were together so I couldn’t receive the calls because he would know what was going on,* ***(Female, 24-year-old, married***)

*I realized I had a challenge when I was in class six*, *I asked my mother if my father knows that I am on care*, *then her answer was that he doesn’t know about it but this can’t deter me from taking the drugs but I should just take them*. *When he [Navigator] sends a text when my father is around*, *I don’t reply back until the following day when he will not be around that’s at noon that is when I reply or call back*. *(****Female*, *15-year-old*, *single***).

Additionally, several AYAH liked that the Navigators called to find out their general well-being, talk about their relationships, family planning and prevention of COVID-19. This showed that the Navigators were not only focusing on HIV care but also other aspects of their lives.

*There is a time, he called inquiring if I had been vaccinated for COVID-19, I told him that I hadn’t, then he told me that it was advisable if I could be vaccinated* ***(Female, 20-year-old, married***)

*To do with relationships*, *they advise what to do when having sex*, *how to protect yourself*, *if you have a baby*, *control measures to be taken* ***(Male*, *18-year-old*, *single***).

One AYAH also felt that there was need to include in the messages other useful information based on what issues are prevailing in the communities. For example, one AYA felt that discussions on hygiene should be included as part of the content of the messaging package. She thought this would be helpful in prevention of the spread of COVID-19 virus.

*The content that should be included is that on hygiene. At the moment we have the Coronavirus, you should include that, encouraging people, giving information, when at home, do you have water for washing hands at your doorstep, when you have a visitor, they first wash their hands before they get in [house]…* ***(Female, 20-year-old, married***).

#### Tone of voice in text messages and calls

All participants reported that the Navigators used a polite, friendly, and an encouraging tone in their conversations with the adolescents and young adults eliciting trust and openness as illustrated below.

*It’s not that scaring, it’s always a friendly tone, whereby I am open to him, and we share. It is motivating too* ***(Male, 21-year-old, single***)

*It’s good in that [name omitted] talks politely*, *happily*, *serious*, *meaning what they intend to for your discussions…* ***(Female*, *20-year-old*, *married***)

#### Enhancing adherence to clinic appointment and ART medication

Overall, AYAH found electronic navigation to be useful in reminding them to keep their clinic appointments and taking ART. The reminders came up during their routine conversations with the navigators, who emphasized the benefits of adhering to ART and keeping clinic appointments.

*It has helped, because she always reminds me of the time that I should go back to the clinic, the time when I am supposed to take my medication, she always asks if I am doing that, yeah, it is a reminder, it’s helpful. At least now each and every time they’ll remind you to always keep the time for taking medication (****Male, 20-year-old, single***)

*I haven’t missed on my clinic visits*, *though they remind us to never miss clinic visits*. ***(Male*, *23-year-old*, *singl***e)

Notably, AYAH with tendencies to forget to take their ART or attend clinic appointments or who expressed fear of going for their clinic visit after missing their appointments found the intervention to be helpful. This is because Navigators reminded them of their clinic date and time and were present at the clinic to offer the necessary support.

*It has helped me, before I used to forget clinic appointment dates, my mother could only remember hers, she has so much going through her mind, so I was the one to remind her and at times I could forget, when we realize that we were supposed to come back to the clinic, they could quarrel at us, why are we not keeping the appointment, but nowadays when it is a week to the date of appointment, they remind me that ‘you are supposed to come for the program, and we are concerned about your health, we are here to help you.* ***(Female, 15-year-old, single***)

*Before I couldn’t take medication even for a week*, *I used to think that I don’t have the disease*, *after some time*, *when I went for a viral load test*, *it was about 274*, *that is when I joined the study*, *since then I proved that I was still having it*, *[laughing] let me just continue with medication*. *I had defaulted and the very day they saw my results was when I joined the E-NAV arm*. *I was being advised as I continue with medication*, *they told me that they didn’t want to see such a viral load*, *they need a zero viral load*. *I was motivated and ever since I joined the study*, *I haven’t missed taking my medication*. ***(Female*, *20-year-old*, *married***)

### Relationship and trust building

Overall participants expressed that the intervention was helpful in building trust with the Navigator. Four participants reported that they could freely share the challenges they had regarding HIV care and treatment without fear of confidentiality breech since some of them were not known to the Navigator.

*I trust her. I know she can’t share my secrets because she doesn’t know me much, we just met here, and she only has my number. (****Female, 21-year-old, married***)

Participants who were known to the Navigator before the study felt they had already built trust and were comfortable discussing their issues with the navigators. These Navigators were previously employed at these study sites.

*I trusted them because we had met, interacted with them before, and discussed a lot [stresses], they know everything about me, that’s why I trusted them that much* ***(Female, 23-year-old, single***)

*I trusted them reason being*, *I was already used to them*. *(****Female*, *23-year-old*, *single***)

However, it was difficult for some participants to build trust at the beginning with the Navigators. The friendliness and approachable attitude of the Navigators, as they continued to interact with participants, made it easier for the participants to open up.

*I didn’t trust them fully, because I wasn’t sure if they’d disclose my information, and even if it were disclosed, I’m okay at the moment. I wasn’t sure because they can say its confidential and a time can come whereby, they share the story with someone else. What made me talk more and trust him was the way he was friendly and his approach. While at the clinic, he called me, we talked as my supporter, and it’s from then we continued interacting. (****Male, 21-year-old, singl***e)

AYAH-navigator relationships were described as fraternal, client-focused, and helped build personal connection. This played a role in motivating AYAH to stay engaged in HIV care and treatment by promoting adherence to ART.

*I was supposed to be taken for an operation and when I shared with them, they asked about how I’d get finances. That is when I realized that they were concerned about my health (****Female, 19-year-old, married***)

*I thought it would invade my privacy initially*, *we started getting used to each other gradually and I came to understand that it’s all about some discussion*, *motivation*, *encouragement (****Male*, *15-year-old*, *single***)

### Building HIV knowledge and managing stigma

Most of the participants reported that the intervention positively impacted their ability to continue engaging in HIV care and treatment through the knowledge they acquired from the Peer Navigators. They learned about ART side effects, prevention approaches, how to handle stigma, and developed skills in communicating their needs.

*It has helped me in that I can manage the stigma, those who underestimate me, I can now stand firm, and say it’s something in you, it’s not affecting you, defiling you, it’s just part of you but it’s not controlling you, but you can control these things, in fact you are just better than others because it’s not a killer disease like the other ones, you are strong, if you keep on taking your drugs you’ll be okay. (* ***Male, 19-year-old, single*** *)*

*To do with relationships*, *they advise what to do when having sex*, *how to protect yourself*, *if you have a baby*, *control measures to be taken*. *It has supported me in relationships*, *that is partly*, *when you visit the hospital they can provide condoms*, *and it is upon you to use them for prevention measures*, *it helps in prevention of so many things such as pregnancy*, *such like*. *(****Male*, *18-year-old*, *single***)

### Navigating at the health clinic

Several participants said that the intervention was helpful in improving their access in the healthcare system. The Navigator served as a link between the participant and the healthcare providers and helped clients who required access to other services. For instance, whenever an AYAH could miss their appointment, they could reach out to the Navigator who would inform the healthcare team.

*I have seen changes because I used to miss clinic appointments and when I came, they could quarrel at me, since we began communicating even if I miss, I know they will tell the concerned people, those who take records and the nurses that I missed because of this and that, she will be coming on this day or that. (****Female, 21-year-old, married***)

AYAH not able to attend clinic could send their caregivers for medication pick up on their behalf by notifying the Navigators.

*Like on 11th [clinic appointment date] I was busy, and I sent my mother on my behalf, so she assisted her when she came in a good way, I told my mother to specifically look for her. (****Male, 21-year-old, singl***e)

## Discussion

Our study documented several mechanisms by which phone-based peer navigation serves as an adjunct to HIV care and treatment with the potential to improved health outcomes among individuals living with HIV. In this study, we explored the experiences of AYAH who received phone-based peer navigation to address barriers to HIV care engagement and ensure viral suppression. Our investigation revealed five key thematic areas including intervention acceptability, enhancing adherence to clinic appointment and ART medication, relationship and trust building, HIV knowledge acquisition and stigma management, and clinic navigation.

Intervention acceptability enhances the probability of successful implementation, adoption and achieving desired outcomes [32]. Various factors including content and context influence intervention acceptability [33]. In the current study, there was a high acceptability of the phone-based peer navigation among study participants. This positive response was attributed to intervention characteristics such as its content, timing, frequency, tone, and the flexible way the intervention was delivered. The intervention content, which was delivered bi-weekly for the text messaging component and monthly for electronic navigation on a preferred day and time by AYAH, primarily focused on exploring and addressing barriers to ART adherence, the importance of clinic attendance, and HIV prevention. It is worth noting that topical issues such as COVID-19 and general health issues were also addressed during these sessions. AYAH identify increased HIV treatment knowledge as a pivotal component in their HIV care and retention, with a lack of such knowledge being associated with poor health outcomes [2]. These findings align with similar findings acknowledging that substantive content provided through digital HIV care navigation influences its acceptability [20]. Furthermore, our study participants accepted the planned frequency of the intervention with the option of contacting the phone-based navigator as needed. The frequency of mHealth interventions is variable, and the most effective frequency is still unknown [34]. Moreover, the flexibility in delivering our intervention was pivotal in its acceptability. The intervention was delivered not only during the weekdays but also extended into the night and weekends to accommodate the schedules of the AYAH. This adaptability allowed the navigators to flex their timing to best meet the needs of AYAH. In real-world setting where facilities typically operate on an 8am to 5pm basis, flexibility of delivering this intervention will be crucial to accommodate the schedules and needs of AYAH. This flexible approach is essential for overcoming barriers to care and improving outcomes for this vulnerable population. While our study indicates a generally high acceptance of the phone based navigation among AYAH, it is important to acknowledge that some studies have reported barriers to acceptability of electronic interventions citing concerns about privacy, confidentiality, lack of understanding on how to use technology, data costs, and limited access to the internet [35, 36]. Additional research may further explore effectiveness of these electronic interventions and which groups of AYAH benefit the most [37].

Prior to implementation of this intervention, we engaged AYAH in designing the intervention and this involvement likely contributed to the high acceptability observed [28]. End-user involvement in intervention design has increasingly become standard in public health innovations, particularly when serving groups such as young people who are not automatically highly engaged in health services. The overall high level of engagement with the platform may reflect the contributions of engaging AYAH when designing intervention content and delivery aimed at improving AYAHs health [38]. Engaging AYAH during the design stage consequently impacts intervention acceptability thereby influencing favorable outcomes [39]. Overall, public health should evolve from focusing exclusively at providing services at scale, to finding ways in which services can be reflexive, responsive and nuanced—all within constraints—to meet the needs of more people [40].

Common barriers to HIV care and treatment among AYAH, such as forgetting to take medication, lack of motivation, medication side effects and structural barriers such as lack of transport to attend clinic, fear of disclosure and stigma have been documented in the literature [41–46]. In the present study, AYAH reported that phone-based peer navigation improved their adherence to clinic appointments and ART medication, especially among those prone to forgetfulness. These helpful reminders were integrated into their routine conversations with the navigators prompting them to attend clinic as scheduled or send their treatment supporters and motivating them to continue taking their ARVs. Furthermore, AYAH reported that the peer navigators were helpful in improving their access to other services within the healthcare system. These findings underscore how peer support is a motivating factor, reinforcing self-care practices and contributing to the development of self-efficacy in HIV care and treatment [47]. Our findings are consistent with other studies that highlight the value of appointment reminders in improving retention among people living with HIV [48–50]. We hypothesize that the relationship developed between the AYAH and the Navigator made the reminders more impactful than just sending the text messages which require less resources.

Peer navigators are uniquely positioned to break barriers between patients and the healthcare system [3]. This crucial connection is established through cultivation of trust and creation of a collaborative working relationship [51] which in some cases is influenced by prior experiences. Engaging with someone online rather than in person can be a challenge, however this type of interaction may be normalized among AYA who have entered adolescence while mobile phones and the use of social media are widespread [52]. However, in the present study, a successful relationship was built after one in-person meeting. AYAH reported that the phone-based navigation allowed them to establish trust with their navigators and freely shared the challenges they were experiencing. These challenges extended beyond HIV care and treatment and included personal matters as well. It is possible that this trust -building process led to a deep personal connection and relationship between the AYAH and navigators subsequently creating a renewed and intensified motivation to continue engaging in HIV care and treatment. Our study alongside previous research, identifies trust and establishment of a collaborative relations as key factors in AYAH-Navigator relationship [53–55]. AYAH Navigators are equipped to provide age-appropriate and tailored support. Their ability to build strong connections with the young participants who are their peers make them distinct from adults who may not possess the skills required to meet the specific needs of this population. Phone based navigation by peers also living with HIV offers a valuable opportunity for dissemination of relevant HIV information in a stigma free environment [56, 57].

AYAH reported notable improvements in their HIV knowledge through their routine interactions with the peer navigators. Majority reported enhanced understanding on ART side effects, stigma management and HIV prevention approaches. Furthermore, they reported having developed skills in effectively communicating their needs, especially during their routine clinic visits. Lack of HIV knowledge and stigma has been associated with poor health seeking behaviors hence impeding HIV reduction efforts [58, 59].

Compared to non-AYAH populations, we observed unique preferences in phone-based navigation, particularly concerning the content of the messages. For example, in a study using mobile communication for reminders and a call back feature among adults living with HIV in Western Kenya, the phone communication was well-received for both preventive and social support reasons [60]. Also, the use of coded language in text messages guarded against accidental disclosure, preventing unintended consequences among AYAH. However, in non-AYAH populations, such as patients with cancer receiving electronic navigation, the message content is typically more straightforward and focused on treatment adherence, symptom management, and appointment reminders, without the same level of emphasis on confidentiality and privacy concerns [61, 62].

Importantly, our intervention offered an opportunity for the AYAH to access support services without needing to be physically present at the health facilities. By leveraging on technology, we may have reduced barriers such as transportation issues, long travel time, the need to take time off school or work thereby making it easier for AYAH to stay engaged with their health care and adhere to their treatment schedules. The use of phone-based navigation is potentially a cost-effective mode of delivery, as it not only minimizes logistical challenges for patients but also enhances the efficiency of health care providers by reducing demands for in-person visits and streamlining follow-up processes [63].

We used a diverse group of adolescents and young adults representing two categories of age groups i.e., 15–19 and 20–24 years and varied socioeconomic backgrounds to elicit their experiences with the phone-based peer navigation. This diversity enhances generalizability of our findings to a wider population. Additionally, thematic analysis employed to identify common themes and patterns in the data provided a systematic and rigorous analysis of the data. We acknowledge that this study is not without limitations. Some of the study participants were already known to the phone-based peer navigators before the start of the intervention since the navigators were earlier employed in the specific study sites where AYAH received HIV care. This pre-existing relationship would potentially introduce social desirability bias into the intervention and could have influenced Navigator-participant relationship due to their previous interactions. Our study included participants who were already experienced and sensitized to the phone-based peer navigation which could potentially introduce positive bias towards the intervention. However, we feel it is important to obtain perspectives from AYAH who experience the intervention and did obtain input from different AYAH who had not yet experienced E-NAV prior to study implementation.

Despite these limitations, the phone-based intervention has shown promising acceptability among AYAH, indicating that it could be a viable method to support this population on a larger scale. However, if the National AIDs and STI Control Program (NASCOP) in Kenya is planning to scale-up this intervention to various counties, it should consider funding, human resources, and systems for outcome evaluation. Given the initial financial investment required, NASCOP can collaborate with phone and internet providers and implementing partners to leverage on their on-site presence and expertise for effective implementation.

## Conclusion

Our findings add evidence to existing literature on acceptability and the role of technology-based interventions in enhancing HIV care engagement among AYAH through trust and successful relationship building. Furthermore, understanding end-user experiences with an intervention provides an opportunity for feedback and making refinements that can lead to sustained positive effects. Given the present challenges with in-person navigation, including AYAH’s inability to attend sessions due to transport costs, school schedules, and stigma, adoption of phone-based peer navigation offers a promising solution for addressing the unique needs of AYAH, with reduced resource requirements and increased flexibility in care delivery. Furthermore, phone-based peer navigation provides important psychosocial and treatment support that may result in positive HIV outcomes among AYAH. Future research needs to consider determining the efficacy of phone-based peer navigation to inform scale-up.

## Supporting information

S1 Text PLOS inclusivity in global research. (DOC)

## References

[1] Mizuno Y, Higa DH, Leighton CA, Roland KB, Deluca JB, Koenig LJ. Is HIV patient navigation associated with HIV care continuum outcomes? AIDS. 2018 Nov 13;32(17):2557–71. doi: 10.1097/QAD.0000000000001987 30102661 PMC6724743

[2] Clair-Sullivan NS, Mwamba C, Whetham J, Moore CB, Darking M, Vera J. Barriers to HIV care and adherence for young people living with HIV in Zambia and mHealth. mHealth [Internet]. 2019 Sep 30 [cited 2022 Aug 29];5(0). Available from: https://mhealth.amegroups.com/article/view/29871 10.21037/mhealth.2019.09.02 PMC6789205 31620472

[3] Gabster A, Socha E, Pascale JM, Talavero GC, Castrellón A, Quiel Y, et al. Barriers and facilitators to antiretroviral adherence and retention in HIV care among people living with HIV in the Comarca Ngäbe-Buglé, Panama. PLOS ONE. 2022 Jun 16;17(6):e0270044. 35709223 10.1371/journal.pone.0270044 PMC9202867

[4] Truong HHM, Guzé MA, Kadede K, Amboka S, Otieno B, Odhiambo H, et al. HIV INFECTION AMONG ADOLESCENTS RESIDING IN URBAN INFORMAL SETTLEMENTS OF KENYA. AIDS Educ Prev. 2023 Jun;35(3):225–34. doi: 10.1521/aeap.2023.35.3.225 37410374 PMC10624479

[5] Onyango DO, Sande MAB van der, Musingila P, Kinywa E, Opollo V, Oyaro B, et al. High HIV prevalence among decedents received by two high-volume mortuaries in Kisumu, western Kenya, 2019. PLOS ONE. 2021 Jul 1;16(7):e0253516. 10.1371/journal.pone.0253516 PMC8248726 34197509

[6] Waruru A, Wamicwe J, Mwangi J, Achia TNO, Zielinski-Gutierrez E, Ng’ang’a L, et al. Where Are the Newly Diagnosed HIV Positives in Kenya? Time to Consider Geo-Spatially Guided Targeting at a Finer Scale to Reach the “First 90.” Frontiers in Public Health [Internet]. 2021 [cited 2022 Jul 20];9. Available from: https://www.frontiersin.org/articles/10.3389/fpubh.2021.503555 10.3389/fpubh.2021.503555 PMC8102680 33968864

[7] Njuguna IN, Beima-Sofie K, Mburu CW, Mugo C, Neary J, Itindi J, et al. Adolescent transition to adult care for HIV-infected adolescents in Kenya (ATTACH): study protocol for a hybrid effectiveness-implementation cluster randomised trial. BMJ Open. 2020 Dec 2;10(12):e039972. doi: 10.1136/bmjopen-2020-039972 33268417 PMC7713196

[8] Nglazi MD, Kranzer K, Holele P, Kaplan R, Mark D, Jaspan H, et al. Treatment outcomes in HIV-infected adolescents attending a community-based antiretroviral therapy clinic in South Africa. BMC Infectious Diseases. 2012 Jan 25;12(1):21. doi: 10.1186/1471-2334-12-21 22273267 PMC3295677

[9] Firdu N, Enquselassie F, Jerene D. HIV-infected adolescents have low adherence to antiretroviral therapy: a cross-sectional study in Addis Ababa, Ethiopia. Pan African Medical Journal [Internet]. 2017 Aug 8 [cited 2024 May 8];27(1). Available from: https://www.ajol.info/index.php/pamj/article/view/159905 10.11604/pamj.2017.27.80.8544 PMC5554655 28819501

[10] Hlophe LD, Tamuzi JL, Shumba CS, Nyasulu PS. Barriers and facilitators to anti-retroviral therapy adherence among adolescents aged 10 to 19 years living with HIV in sub-Saharan Africa: A mixed-methods systematic review and meta-analysis. PLOS ONE. 2023 May 18;18(5):e0276411. doi: 10.1371/journal.pone.0276411 37200399 PMC10194875

[11] Traynor SM, Schmidt RD, Gooden LK, Matheson T, Haynes L, Rodriguez A, et al. Differential Effects of Patient Navigation across Latent Profiles of Barriers to Care among People Living with HIV and Comorbid Conditions. Journal of Clinical Medicine. 2023 Jan;12(1):114. 10.3390/jcm12010114 PMC9820894 36614917

[12] Freeman HP, Rodriguez RL. History and principles of patient navigation. Cancer. 2011;117(S15):3537–40. doi: 10.1002/cncr.26262 21780088 PMC4557777

[13] Greene GJ, Reidy E, Felt D, Marro R, Johnson AK, Phillips G, et al. Implementation and evaluation of patient navigation in Chicago: Insights on addressing the social determinants of health and integrating HIV prevention and care services. Evaluation and Program Planning. 2022 Feb 1;90:101977. doi: 10.1016/j.evalprogplan.2021.101977 34373116

[14] Karver TS, Barrington C, Donastorg Y, Perez M, Gomez H, Page KR, et al. Exploring peer navigation and support in the quality of HIV care experiences of female sex workers in the Dominican Republic. BMC Health Serv Res. 2022 Dec;22(1):1–11. 35016659 10.1186/s12913-021-07439-4 PMC8753897

[15] Geng EH, Odeny TA, Montoya LM, Iguna S, Kulzer JL, Adhiambo HF, et al. Adaptive Strategies for Retention in Care among Persons Living with HIV. NEJM Evidence. 2023 Mar 28;2(4):EVIDoa2200076. doi: 10.1056/evidoa2200076 38143482 PMC10745095

[16] Muessig KE, Nekkanti M, Bauermeister J, Bull S, Hightow-Weidman LB. A Systematic Review of Recent Smartphone, Internet and Web 2.0 Interventions to Address the HIV Continuum of Care. Curr HIV/AIDS Rep. 2015 Mar;12(1):173–90. doi: 10.1007/s11904-014-0239-3 25626718 PMC4370788

[17] Hausmann JS, Touloumtzis C, White MT, Colbert JA, Gooding H. Adolescent and Young Adult Use of Social Media For Health and its Implications. J Adolesc Health. 2017 Jun;60(6):714–9. doi: 10.1016/j.jadohealth.2016.12.025 28259620 PMC5441939

[18] Marcolino MS, Oliveira JAQ, D’Agostino M, Ribeiro AL, Alkmim MBM, Novillo-Ortiz D. The Impact of mHealth Interventions: Systematic Review of Systematic Reviews. JMIR mHealth and uHealth. 2018 Jan 17;6(1):e8873. doi: 10.2196/mhealth.8873 29343463 PMC5792697

[19] Rubin S, Bouri N, Jolani N, Minton K. The Adoption of Social Media and Mobile Health Technologies for Emergency Preparedness by Local Health Departments: A Joint Perspective From NACCHO and the UPMC Center for Health Security. Journal of Public Health Management and Practice. 2014 Apr;20(2):259–63. doi: 10.1097/PHH.0000000000000056 24458314

[20] Arayasirikul S, Turner C, Trujillo D, Le V, Wilson EC. Efficacy and Impact of Digital HIV Care Navigation in Young People Living With HIV in San Francisco, California: Prospective Study. JMIR mHealth and uHealth. 2020 May 8;8(5):e18597. doi: 10.2196/18597 32383680 PMC7244993

[21] Trujillo D, Turner C, Le V, Wilson EC, Arayasirikul S. Digital HIV Care Navigation for Young People Living With HIV in San Francisco, California: Feasibility and Acceptability Study. JMIR Mhealth Uhealth. 2020 Jan 10;8(1):e16838. doi: 10.2196/16838 31922489 PMC6996763

[22] Malik A, Antonino A, Khan ML, Nieminen M. Characterizing HIV discussions and engagement on Twitter. Health Technol. 2021 Nov 1;11(6):1237–45.

[23] Belzer ME, Naar-King S, Olson J, Sarr M, Thornton S, Kahana SY, et al. The Use of Cell Phone Support for Non-adherent HIV-Infected Youth and Young Adults: An Initial Randomized and Controlled Intervention Trial. AIDS and behavior. 2014 Apr;18(4):686. doi: 10.1007/s10461-013-0661-3 24271347 PMC3962719

[24] Garofalo R, Adetunji A, Kuhns LM, Omigbodun O, Johnson AK, Kuti K, et al. Evaluation of the iCARE Nigeria Pilot Intervention Using Social Media and Peer Navigation to Promote HIV Testing and Linkage to Care Among High-Risk Young Men: A Nonrandomized Controlled Trial. JAMA Network Open. 2022 Feb 22;5(2):e220148. doi: 10.1001/jamanetworkopen.2022.0148 35191969 PMC8864509

[25] Kharono B, Kaggiah A, Mugo C, Seeh D, Guthrie BL, Moreno M, et al. Mobile technology access and use among youth in Nairobi, Kenya: implications for mobile health intervention design. Mhealth. 2022 Jan 20;8:7. doi: 10.21037/mhealth-21-23 35178438 PMC8800198

[26] Abuogi LL, Kulzer JL, Akama E, Odeny TA, Eshun-Wilson I, Petersen M, et al. Adapt for Adolescents: Protocol for a sequential multiple assignment randomized trial to improve retention and viral suppression among adolescents and young adults living with HIV in Kenya. Contemp Clin Trials. 2023 Apr;127:107123. doi: 10.1016/j.cct.2023.107123 36813086 PMC10075086

[27] Tenny S, Brannan JM, Brannan GD. Qualitative Study. In: StatPearls [Internet]. Treasure Island (FL): StatPearls Publishing; 2024 [cited 2024 Jun 3]. Available from: http://www.ncbi.nlm.nih.gov/books/NBK470395/

[28] Akama EO, Beres LK, Kulzer JL, Ontuga G, Adhiambo H, Bushuru S, et al. A youth-centred approach to improving engagement in HIV services: human-centred design methods and outcomes in a research trial in Kisumu County, Kenya. BMJ Global Health. 2023 Nov 1;8(11):e012606. 10.1136/bmjgh-2023-012606 PMC10689376 38030226

[29] Eshun-Wilson I, Akama E, Adhiambo F, Kwena Z, Oketch B, Obatsa S, et al. Adolescent and young adult preferences for financial incentives to support adherence to antiretroviral therapy in Kenya: a mixed methods study. J Int AIDS Soc. 2022 Sep;25(9):e25979. doi: 10.1002/jia2.25979 36109803 PMC9478044

[30] Palinkas LA, Horwitz SM, Green CA, Wisdom JP, Duan N, Hoagwood K. Purposeful sampling for qualitative data collection and analysis in mixed method implementation research. Adm Policy Ment Health. 2015 Sep;42(5):533–44. doi: 10.1007/s10488-013-0528-y 24193818 PMC4012002

[31] Braun V, Clarke V. Using thematic analysis in psychology. Qualitative Research in Psychology. 2006 Jan;3(2):77–101.

[32] Sekhon M, Cartwright M, Francis JJ. Development of a theory-informed questionnaire to assess the acceptability of healthcare interventions. BMC Health Services Research. 2022 Mar 1;22(1):279. doi: 10.1186/s12913-022-07577-3 35232455 PMC8887649

[33] Sekhon M, Cartwright M, Francis JJ. Acceptability of healthcare interventions: an overview of reviews and development of a theoretical framework. BMC Health Services Research. 2017 Jan 26;17(1):88. doi: 10.1186/s12913-017-2031-8 28126032 PMC5267473

[34] Finitsis DJ, Pellowski JA, Johnson BT. Text message intervention designs to promote adherence to antiretroviral therapy (ART): a meta-analysis of randomized controlled trials. PLoS One. 2014;9(2):e88166. doi: 10.1371/journal.pone.0088166 24505411 PMC3914915

[35] Feroz AS, Ali NA, Khoja A, Asad A, Saleem S. Using mobile phones to improve young people sexual and reproductive health in low and middle-income countries: a systematic review to identify barriers, facilitators, and range of mHealth solutions. Reproductive Health. 2021 Jan 16;18(1):9. doi: 10.1186/s12978-020-01059-7 33453723 PMC7811742

[36] Bear HA, Nunes LA, Ramos G, Manchanda T, Fernandes B, Chabursky S, et al. The Acceptability, Engagement, and Feasibility of Mental Health Apps for Marginalized and Underserved Young People: Systematic Review and Qualitative Study. Journal of Medical Internet Research. 2024 Jul 30;26(1):e48964. doi: 10.2196/48964 39078699 PMC11322694

[37] Crowley T, Petinger C, Nchendia AI, Wyk B van. Effectiveness, Acceptability and Feasibility of Technology-Enabled Health Interventions for Adolescents Living with HIV in Low- and Middle-Income Countries: A Systematic Review. International Journal of Environmental Research and Public Health. 2023 Jan 30;20(3):2464. 36767831 10.3390/ijerph20032464 PMC9916219

[38] Geng EH, Nash D, Phanuphak N, Green K, Solomon S, Grimsrud A, et al. The question of the question: impactful implementation science to address the HIV epidemic. J Int AIDS Soc. 2022 Apr;25(4):e25898. doi: 10.1002/jia2.25898 35384312 PMC8982316

[39] Mukherjee TI, Zerbe A, Falcao J, Carey S, Iaccarino A, Kolada B, et al. Human-Centered Design for Public Health Innovation: Codesigning a Multicomponent Intervention to Support Youth Across the HIV Care Continuum in Mozambique. Glob Health Sci Pract. 2022 Apr 28;10(2):e2100664. doi: 10.9745/GHSP-D-21-00664 35487546 PMC9053144

[40] Geng EH, Holmes CB, Moshabela M, Sikazwe I, Petersen ML. Personalized public health: An implementation research agenda for the HIV response and beyond. PLOS Medicine. 2019 Dec 31;16(12):e1003020. doi: 10.1371/journal.pmed.1003020 31891581 PMC6938296

[41] MacDonell K, Naar-King S, Huszti H, Belzer M. Barriers to Medication Adherence in Behaviorally and Perinatally Infected Youth Living with HIV. AIDS Behav. 2013 Jan;17(1):86–93. doi: 10.1007/s10461-012-0364-1 23142855 PMC3549030

[42] Macdonell KE, Naar-King S, Murphy DA, Parsons JT, Huszti H. Situational temptation for HIV medication adherence in high-risk youth. AIDS Patient Care STDS. 2011 Jan;25(1):47–52. doi: 10.1089/apc.2010.0172 21162691 PMC3030911

[43] Mulawa MI, LeGrand S, Hightow-Weidman LB. eHealth to Enhance Treatment Adherence among Youth Living with HIV. Curr HIV/AIDS Rep. 2018 Aug;15(4):336–49. doi: 10.1007/s11904-018-0407-y 29959649 PMC6086132

[44] Audi C, Jahanpour O, Antelman G, Guay L, Rutaihwa M, van de Ven R, et al. Facilitators and barriers to antiretroviral therapy adherence among HIV-positive adolescents living in Tanzania. BMC Public Health. 2021 Dec 13;21:2274. doi: 10.1186/s12889-021-12323-1 34903209 PMC8670050

[45] Kwena ZA, Amico RK, Masvawure TB, Ngure KK, Bukusi EA, Remien RH, et al. Barriers to linkage and retention in HIV care still persist among adolescent girls and young women in western Kenya. African Journal of AIDS Research. 2023 Apr 3;22(2):71–84. doi: 10.2989/16085906.2023.2197879 37337818

[46] Yang E, Mphele S, Moshashane N, Bula B, Chapman J, Okatch H, et al. Distinctive Barriers to Antiretroviral Therapy Adherence among Non-adherent Adolescents Living with HIV in Botswana. AIDS Care. 2018 Feb;30(2):224–31. doi: 10.1080/09540121.2017.1344767 28643572 PMC6083824

[47] World Health Organization. HIV and adolescents: guidance for HIV testing and counselling and care for adolescents living with HIV: recommendations for a public health approach and considerations for policy-makers and managers [Internet]. Geneva: World Health Organization; 2013. Available from: https://apps.who.int/iris/handle/10665/94334 25032477

[48] Adams JA, Whiteman K, McGraw S. Reducing Missed Appointments for Patients With HIV: An Evidence-Based Approach. J Nurs Care Qual. 2020;35(2):165–70. doi: 10.1097/NCQ.0000000000000434 31464846

[49] Cornelius JB, Dmochowski J, Boyer C, St Lawrence J, Lightfoot M, Moore M. Text-Messaging-Enhanced HIV Intervention for African American Adolescents: A Feasibility Study. J Assoc Nurses AIDS Care. 2013;24(3):256–67. doi: 10.1016/j.jana.2012.06.005 23122907 PMC3627821

[50] Mehra N, Tunje A, Hallström IK, Jerene D. Effectiveness of mobile phone text message reminder interventions to improve adherence to antiretroviral therapy among adolescents living with HIV: A systematic review and meta-analysis. PLoS One. 2021 Jul 22;16(7):e0254890. doi: 10.1371/journal.pone.0254890 34293033 PMC8297901

[51] Parker VA, Clark JA, Leyson J, Calhoun E, Carroll JK, Freund KM, et al. Patient Navigation: Development of a Protocol for Describing What Navigators Do. Health Serv Res. 2010 Apr;45(2):514–31. doi: 10.1111/j.1475-6773.2009.01079.x 20132342 PMC2838158

[52] Siegel A, Zuo Y, Moghaddamcharkari N, McIntyre RS, Rosenblat JD. Barriers, benefits and interventions for improving the delivery of telemental health services during the coronavirus disease 2019 pandemic: a systematic review. Curr Opin Psychiatry. 2021 Jul;34(4):434–43. doi: 10.1097/YCO.0000000000000714 33928918 PMC8183246

[53] Adeagbo OA, Seeley J, Gumede D, Xulu S, Dlamini N, Luthuli M, et al. Process evaluation of peer-to-peer delivery of HIV self-testing and sexual health information to support HIV prevention among youth in rural KwaZulu-Natal, South Africa: qualitative analysis. BMJ Open. 2022 Feb 14;12(2):e048780. doi: 10.1136/bmjopen-2021-048780 35165105 PMC8845207

[54] Zanoni BC, Sibaya T, Cairns C, Haberer JE. Barriers to Retention in Care are Overcome by Adolescent-friendly Services for Adolescents Living with HIV in South Africa: A Qualitative Analysis. AIDS Behav. 2019 Apr;23(4):957–65. doi: 10.1007/s10461-018-2352-6 30535836 PMC6459720

[55] Wogrin C, Willis N, Mutsinze A, Chinoda S, Verhey R, Chibanda D, et al. It helps to talk: A guiding framework (TRUST) for peer support in delivering mental health care for adolescents living with HIV. PLoS One. 2021;16(3):e0248018. doi: 10.1371/journal.pone.0248018 33657185 PMC7928463

[56] Step MM, Knight K, McMillen Smith J, Lewis SA, Russell TJ, Avery AK. Positive Peers Mobile Application Reduces Stigma Perception Among Young People Living With HIV. Health Promotion Practice. 2020 Sep 1;21(5):744–54. doi: 10.1177/1524839920936244 32757838

[57] Arayasirikul S, Turner CM, Trujillo D, Maycott J, Wilson EC. The Dose Response Effects of Digital HIV Care Navigation on Mental Health and Viral Suppression Among Young People Living With HIV: Single-Arm, Prospective Study With a Pre-Post Design. J Med Internet Res. 2022 Jul 18;24(7):e33990. doi: 10.2196/33990 35849442 PMC9345131

[58] Pampalia N, Waluyo A, Yona S. Knowledge, stigma and health-seeking behavior of patients co-infected with HIV and tuberculosis in Jakarta. Enfermería Clínica. 2021 Apr 1;31:S291–5.

[59] Alemu T, Biadgilign S, Deribe K, Escudero HR. Experience of stigma and discrimination and the implications for healthcare seeking behavior among people living with HIV/AIDS in resource-limited setting. SAHARA J. 2013 Mar;10(1):1–7. doi: 10.1080/17290376.2013.806645 23721543 PMC3819651

[60] Adhiambo HF, Guze M, Kulzer JL, Akama E, Kwena Z, Odeny T, et al. A Descriptive Analysis of Calls and Text Messaging Communication Between Participants and Health Care Providers. In: AIDS RESEARCH AND HUMAN RETROVIRUSES. MARY ANN LIEBERT, INC 140 HUGUENOT STREET, 3RD FL, NEW ROCHELLE, NY 10801 USA; 2018. p. 277–277.

[61] Lazard AJ, Collins MKR, Hedrick A, Varma T, Love B, Valle CG, et al. Using Social Media for Peer-to-Peer Cancer Support: Interviews With Young Adults With Cancer. JMIR Cancer. 2021 Sep 2;7(3):e28234. doi: 10.2196/28234 34473063 PMC8446843

[62] Ronen K, Unger JA, Drake AL, Perrier T, Akinyi P, Osborn L, et al. SMS messaging to improve ART adherence: perspectives of pregnant HIV-infected women in Kenya on HIV-related message content. AIDS Care. 2018;30(4):500–5. doi: 10.1080/09540121.2017.1417971 29254362 PMC5839109

[63] McColl LD, Rideout PE, Parmar TN, Abba-Aji A. Peer support intervention through mobile application: An integrative literature review and future directions. Canadian Psychology / Psychologie canadienne. 2014;55(4):250–7.

